# Supplementary figures and images for: Short-range template switching in great ape genomes explored using pair hidden Markov models
Source: PLoS Genet. 2021 Mar 2;17(3):e1009221. doi: 10.1371/journal.pgen.1009221 (PMC7954356; doi:10.1371/journal.pgen.1009221)

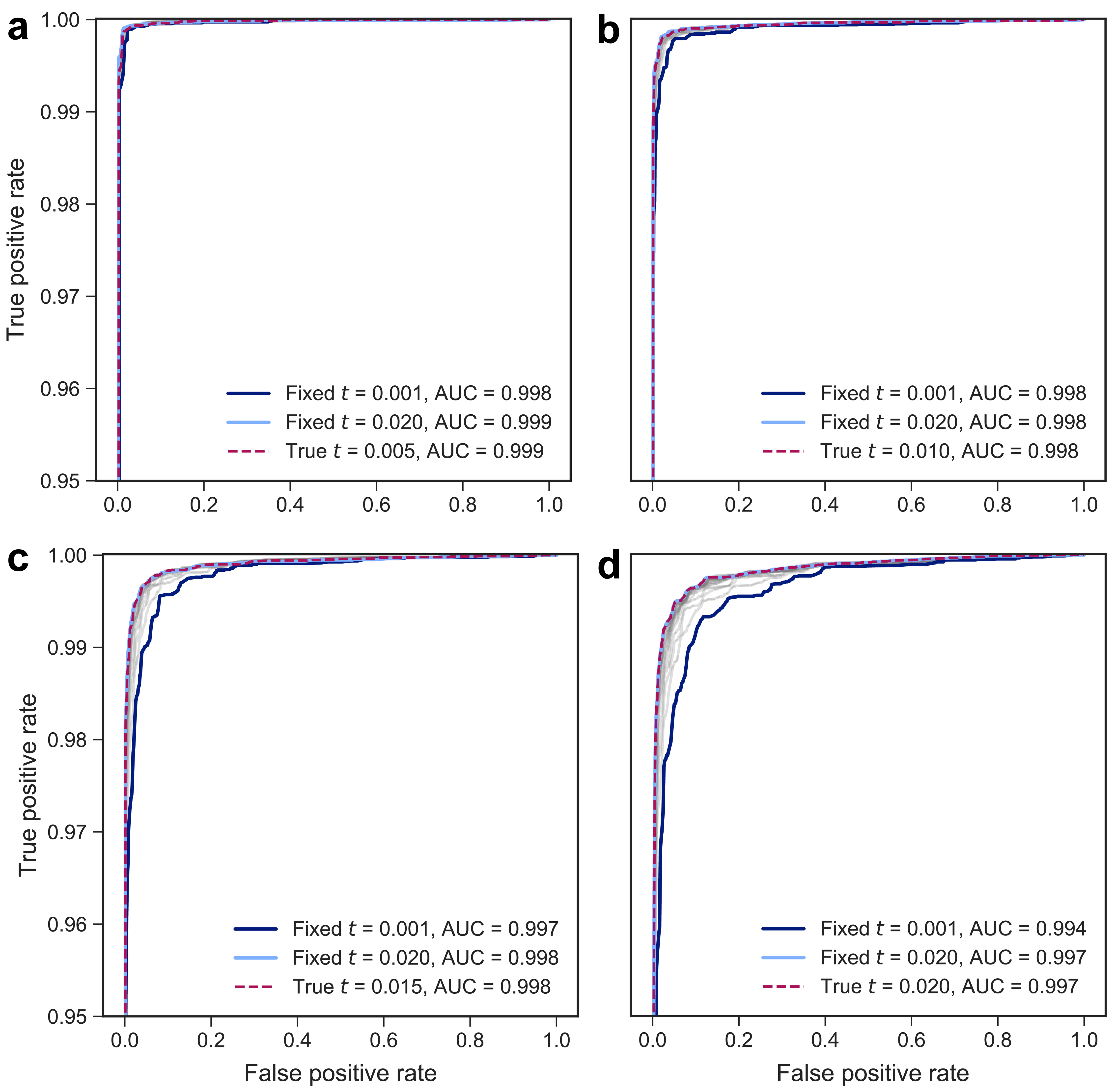

Supplement: S1 Fig — ROC curves for simulations at evolutionary distances of (A) 0.005, (B) 0.010, (C) 0.015, and (D) 0.020. At each evolutionary distance, the TSA pairHMM parameter t was set independently of the evolutionary distance used for sequence simulation, ranging from 0.001 to 0.02 in 0.001 increments. The ROC curve for the t parameter corresponding to the true evolutionary distance is shown as a dashed magenta line, the minimum and maximum fixed t values are in dark blue and light blue, respectively, and all other values of t are shown in grey. Across all fixed evolutionary distances, almost identical performance is achieved using the true t and using the highest fixed value of t, while marginally worse performance is observed when fixing t to smaller values. The performance differences are so small (as measured by the area under the ROC curve (AUC)) that any misspecification of t will have a negligible impact on model performance, indicating that our inferences are robust to our assumed values of t. Note that all y-axes start at 0.95, as the ROC curves between specified values of t would otherwise be indistinguishable. (TIF) [file pgen.1009221.s001.tif]

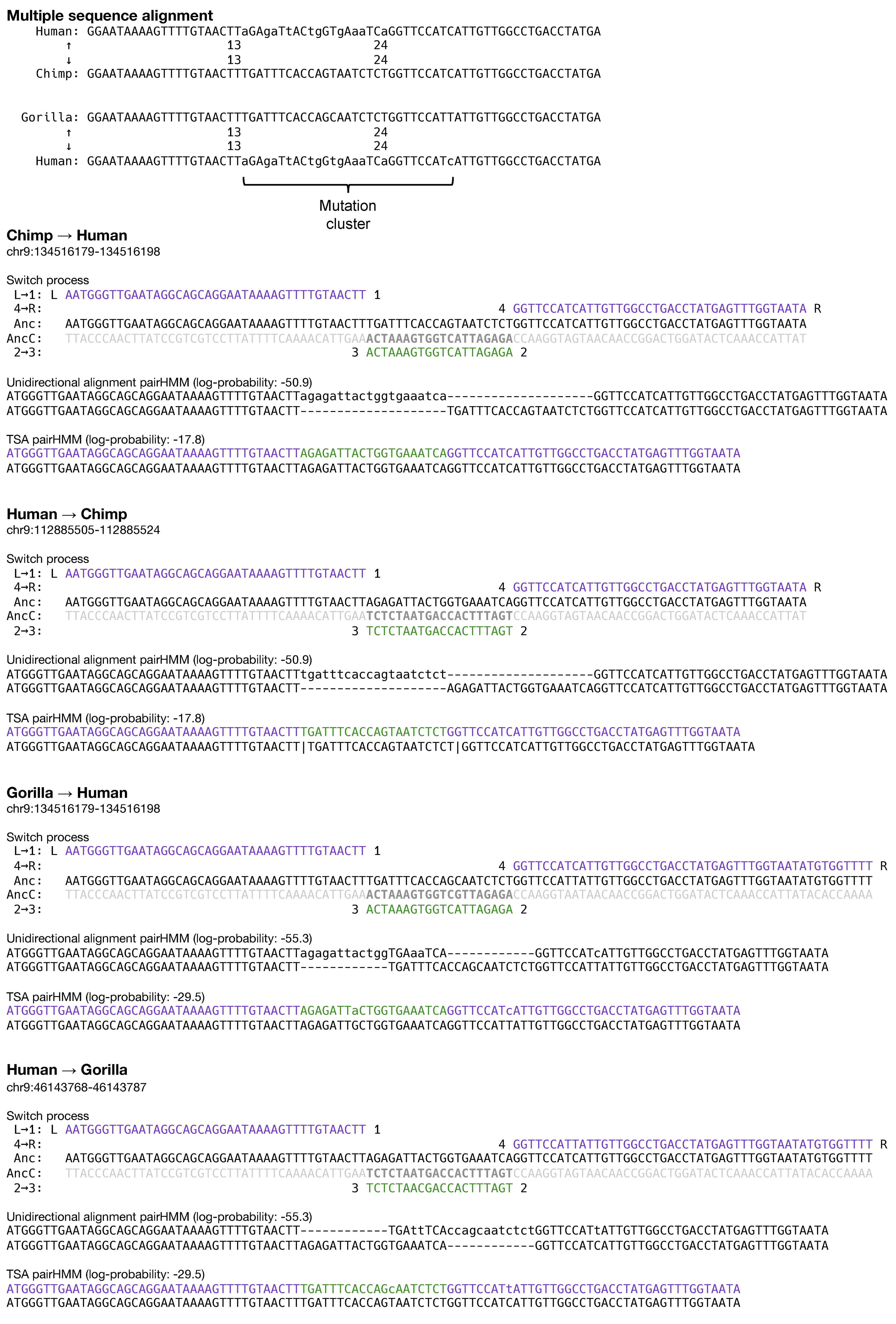

Supplement: S2 Fig — A cluster of mutations is observed between human/chimpanzee and human/gorilla, appearing as either a large cluster of substitutions (input multiple alignment, top), or as a large insertion and deletion event (unidirectional pairHMM alignments). Regardless of which species is specified as the ancestral sequence x or the descendant sequence y, the event is detected as significant (reversible detection; S1 Data, event 3803). As we cannot tell whether this event is congruent with the species tree or represents a region of incomplete lineage sorting, we are unable to place it onto an evolutionary lineage. Coordinates are retrieved from the input Ensembl EPO alignment, and in this case refer to positions from sequences aligned to the negative strand of GRCh38. Note that “Anc” refers to the assumed ancestral sequence and “AncC” refers to the complement of this sequence. (TIF) [file pgen.1009221.s002.tif]

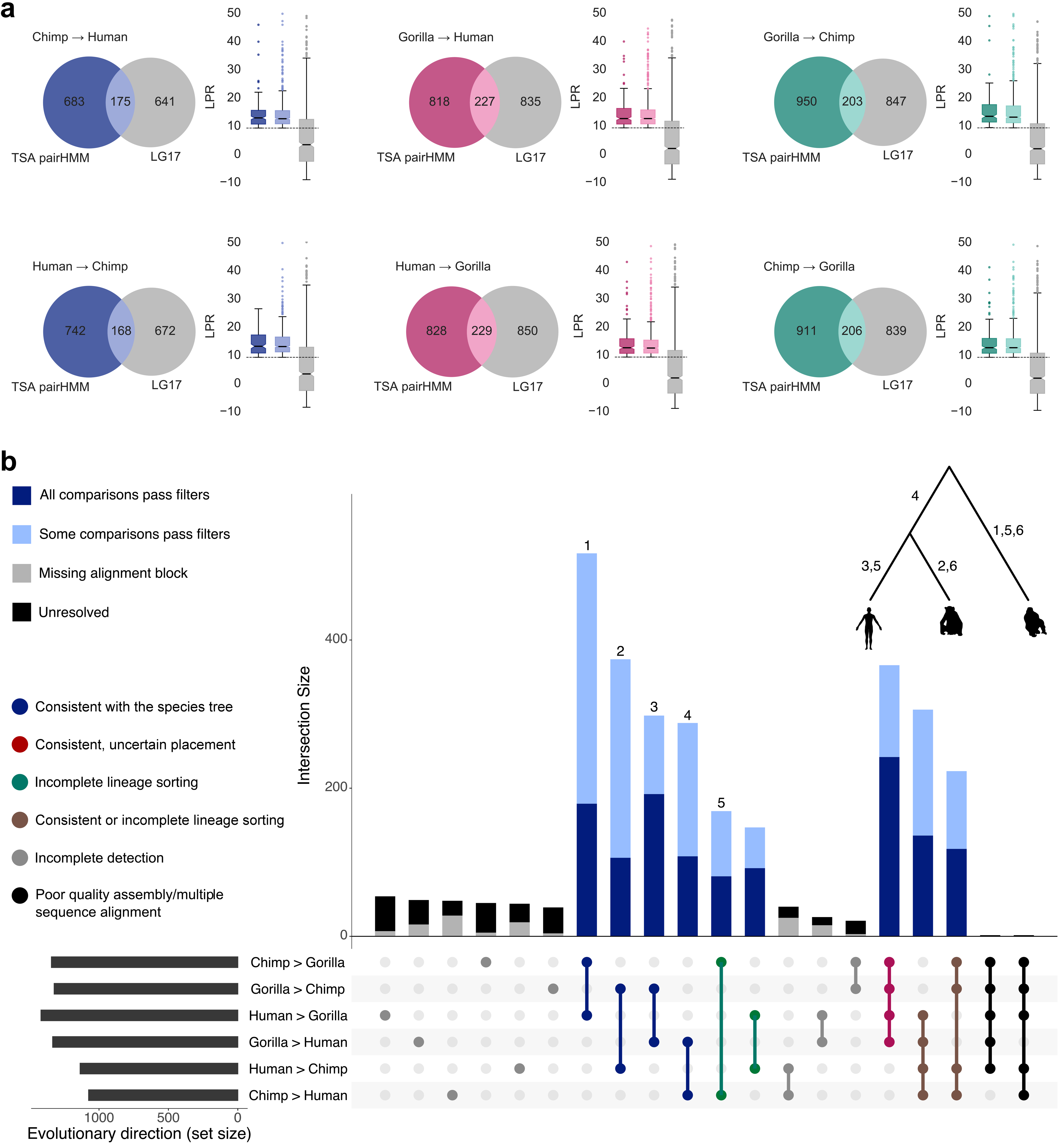

Supplement: S3 Fig — (A) Intersection between the set of template switch events found using the approach of [15], denoted “LG17”, and the significant set of events identified using the TSA pairHMM. Box plots show log-probability ratios for each event set, as well as for candidate events found with both methods. The y-axes are limited to 50 for clarity. (B) Evolutionary direction for the LG17 event set; annotation as in Fig 4, but with an additional category in the dot matrix (shown in black, far right), corresponding to events that are not compatible with a three species tree, likely falling in regions of poor quality sequence assembly or erroneous multiple sequence alignment. (TIF) [file pgen.1009221.s003.tif]

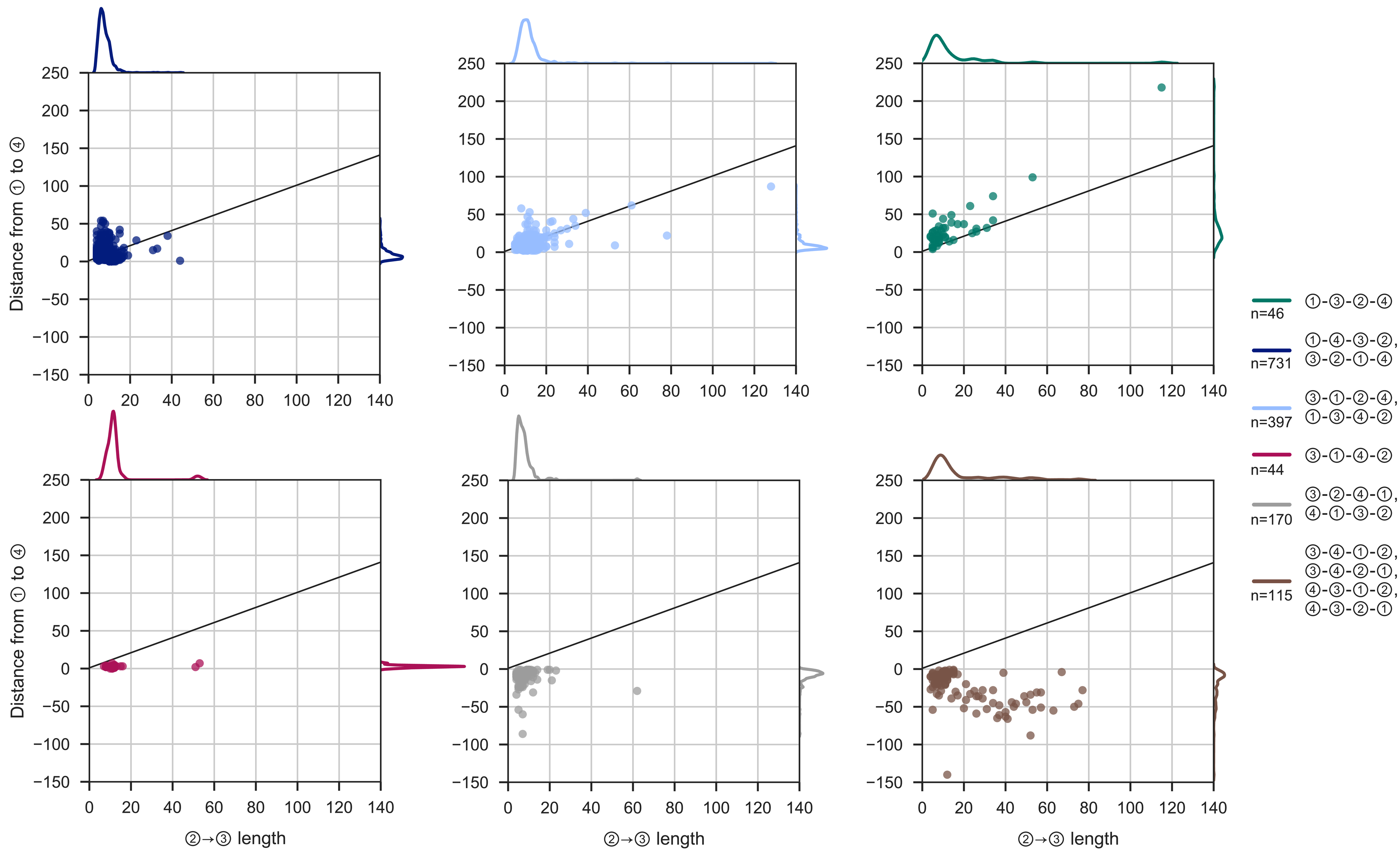

Supplement: S4 Fig — Plots are exactly as in Fig 5A, with the points and marginal densities for the six distinguishable event types shown on separate panels. (TIF) [file pgen.1009221.s004.tif]

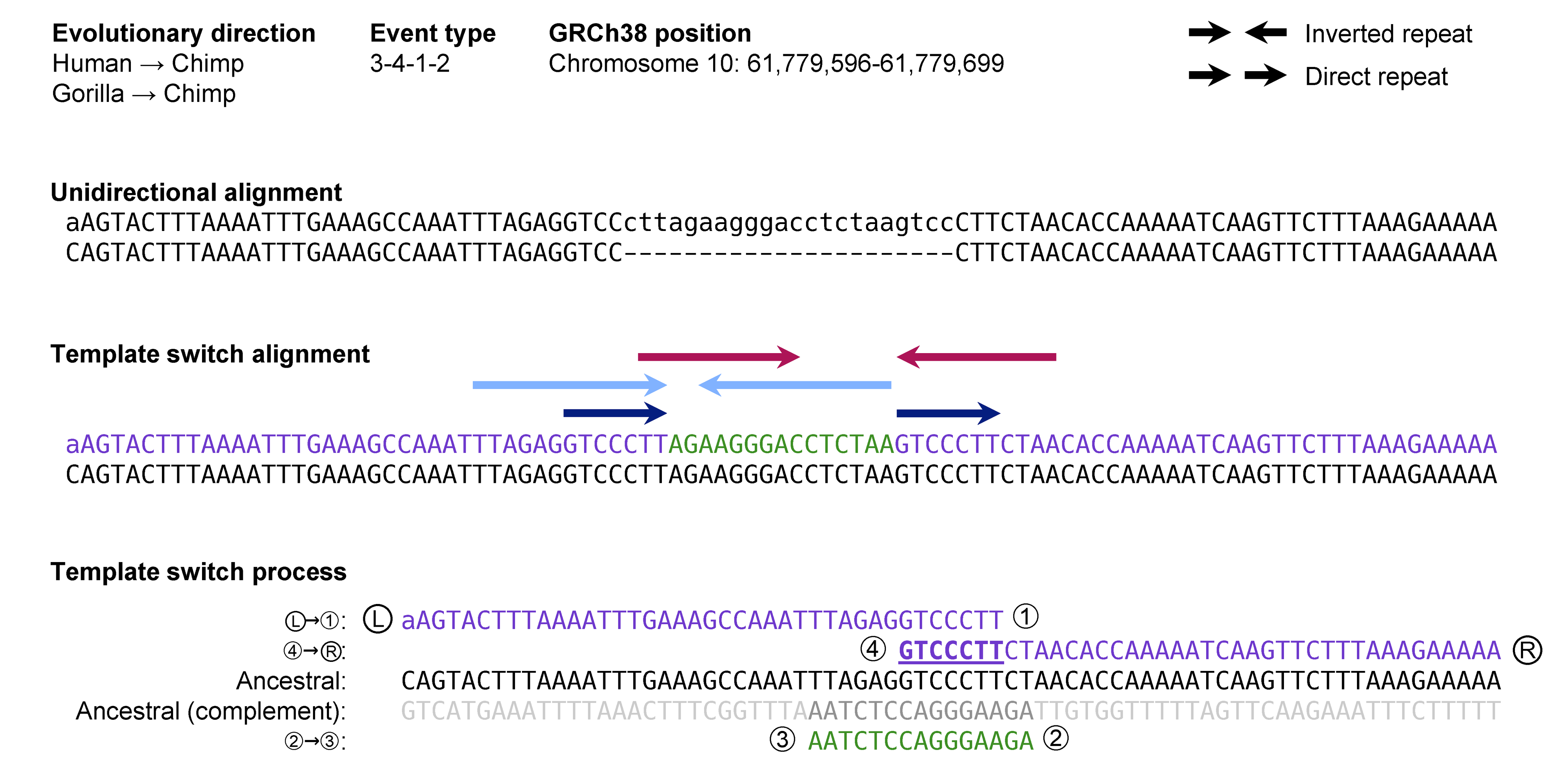

Supplement: S5 Fig — Figure shows, top to bottom, annotation, linear alignment, template-switch alignment and underlying switch process. The bold, underlined region between ④ and ① represents the nascent DNA strand prior to the initial switch event at ①, which typically forms hydrogen-bonded base pairs behind the proceeding replisome, preventing its further involvement in ongoing replication. For events in which ④ precedes ①, a direct repeat generated in the descendant sequence (dark blue arrows above the template-switch alignment) indicates that this region was not sequestered from the replisome through base pairing, and facilitated the final ③ to ④ switch event through an open conformation. The mutational consequence of this event is a complicated rearrangement pattern, manifesting as a series of direct and inverted repeats at the sequence level, shown by coloured arrows above the template-switch alignment (direct repeats shown as arrows in the same orientation; reverse complement regions shown with arrows in opposite orientation). This event is number 145, S1 Data. (TIF) [file pgen.1009221.s005.tif]

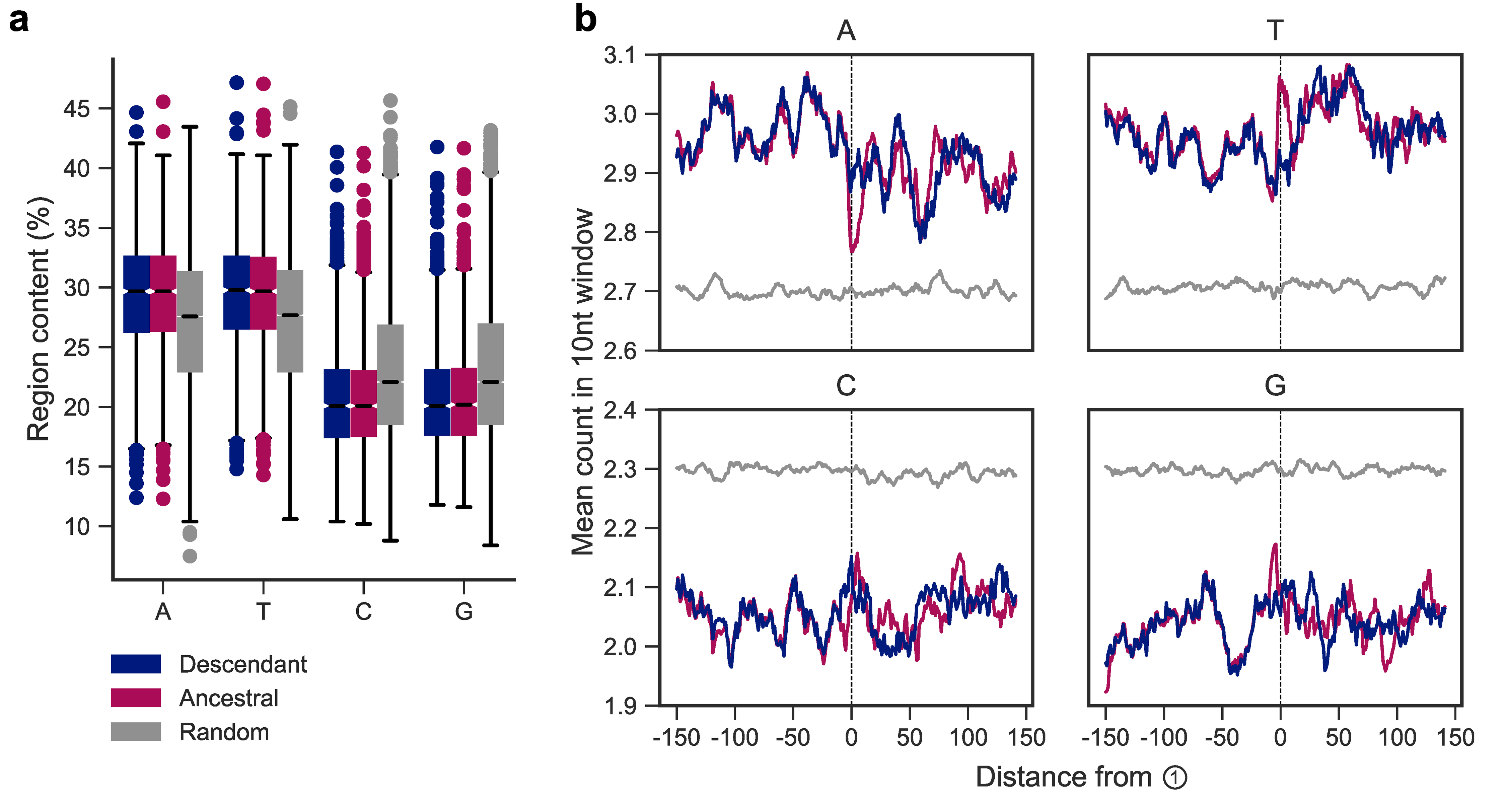

Supplement: S6 Fig — (A) Percentage of each nucleotide in the ancestral and descendant sequence region, compared to a random genomic background. Percentages are calculated in a region ±150nt around ① loci; to form our random background distribution, 10,000 regions of 301nt were randomly drawn from each of the human, chimpanzee, and gorilla genomes. (B) Counts of each nucleotide in a left-aligned single nucleotide sliding window of 10 bases, averaged across descendant, ancestral and randomly sampled sequences at each position. (TIF) [file pgen.1009221.s006.tif]

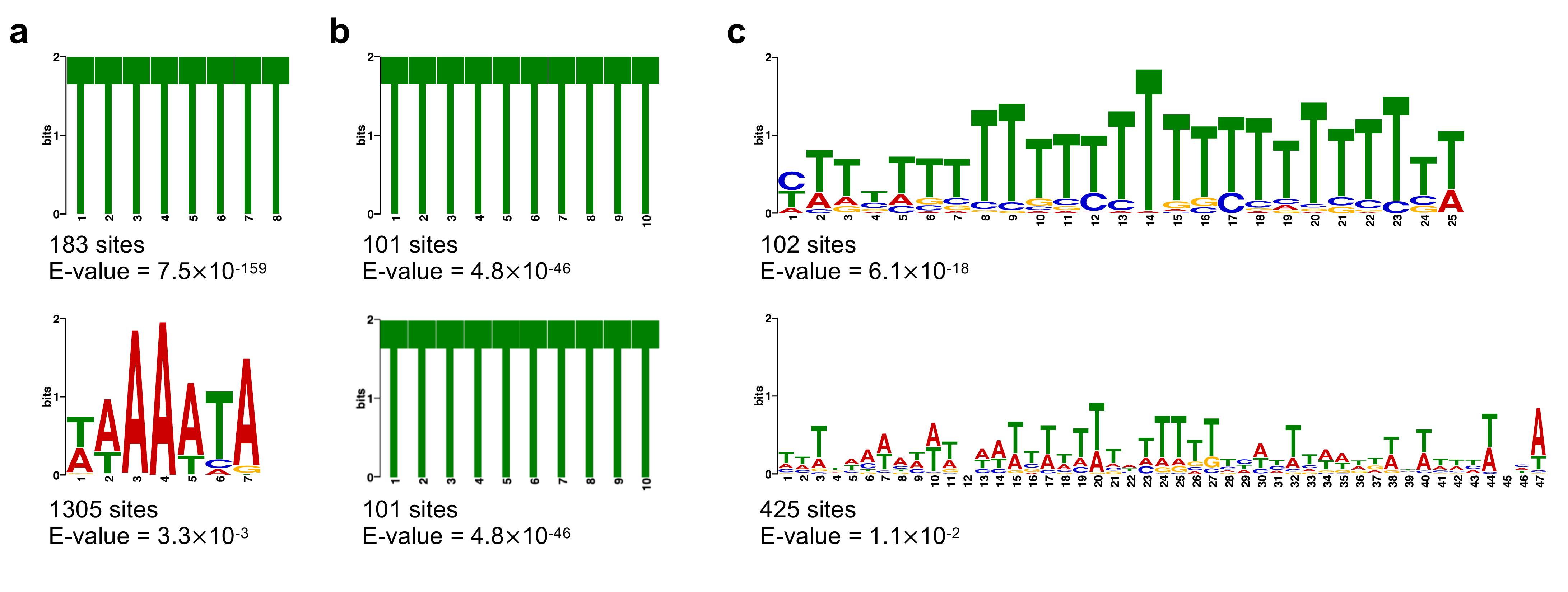

Supplement: S7 Fig — The most significantly enriched motifs (lowest E-value; top row) and most frequent significant motifs (bottom row) within ±150nt of ① for gold-standard events. Motifs were tested for enrichment at three motif size ranges: (A) 6–10nt (B) 10–20nt (C) 20–50nt. In (B), note that for the 10–20nt motif search the same motif (T10) is both most significant and most numerous. (TIF) [file pgen.1009221.s007.tif]

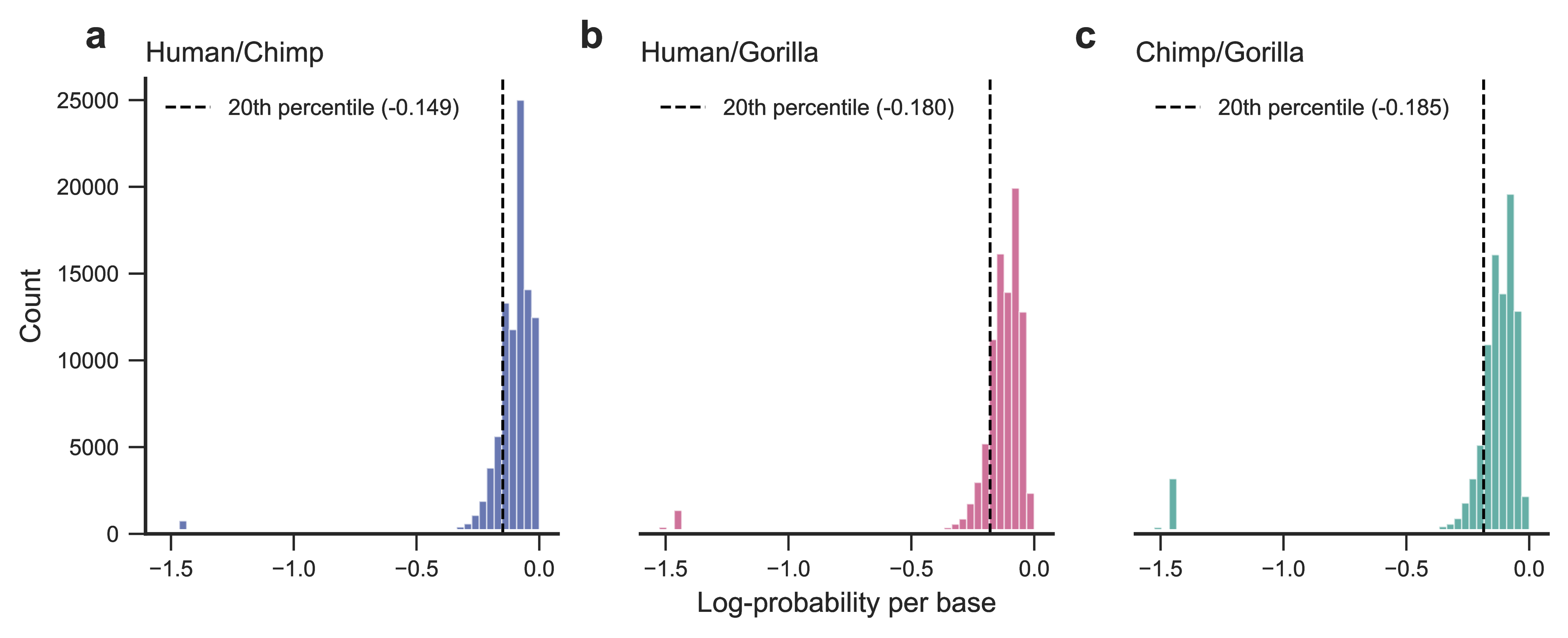

Supplement: S8 Fig — The derived log-probabilities of sampled alignment regions are normalised by final alignment length to produce per-base log-probabilities. Dashed lines represent the 20th percentile thresholds used as baseline alignment quality thresholds for event regions for each pairwise comparison. If both the null model and the template switch model alignments in a region fail this threshold, the region is removed from our analyses. (TIF) [file pgen.1009221.s008.tif]

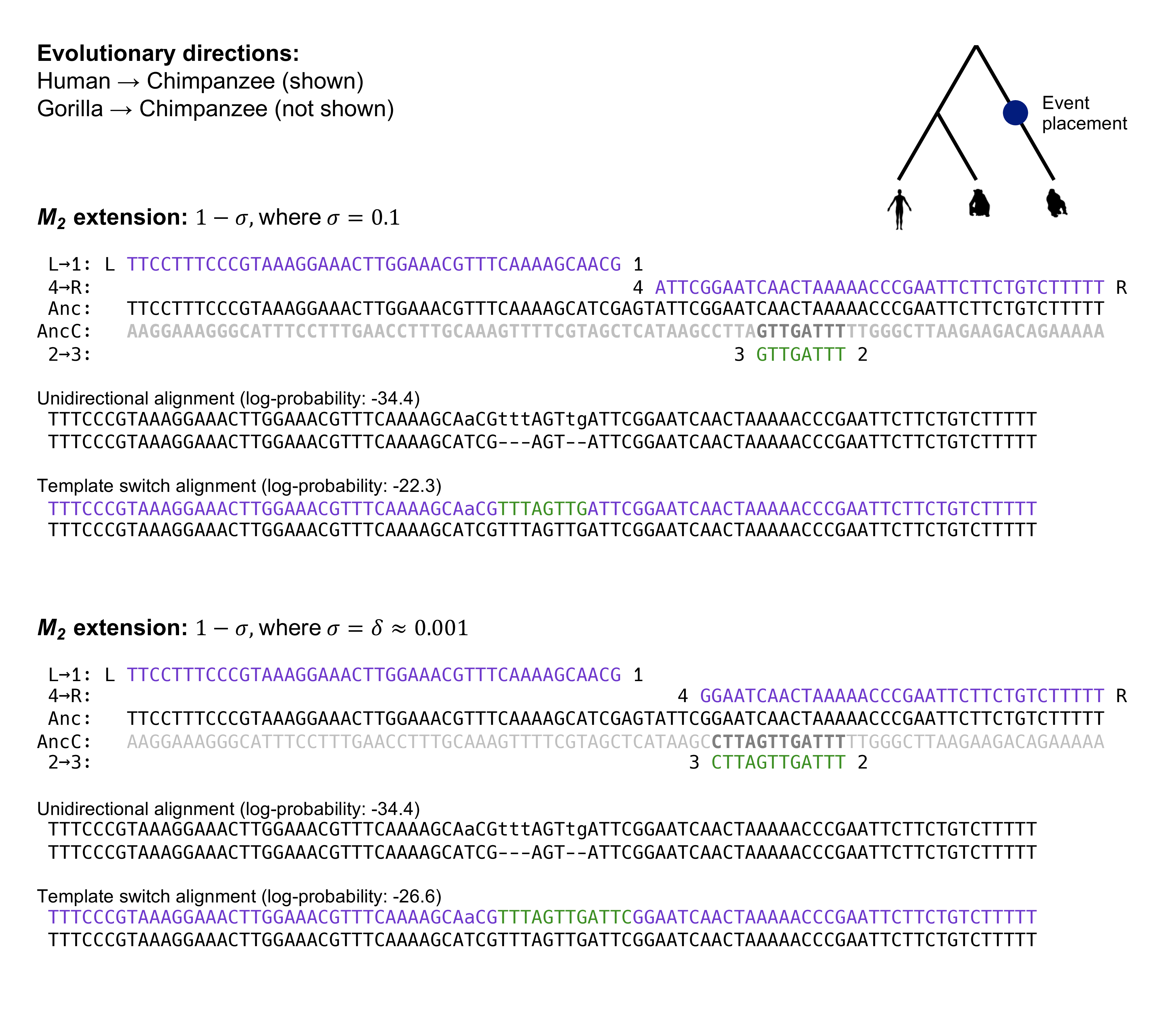

Supplement: S9 Fig — For the chosen value of σ used in the main text (0.1, top), and a nominal small value of sigma (σ = δ = 0.001, bottom), an event detected in the human→chimpanzee and gorilla→chimpanzee directions is shown. When using σ = 0.1, this event does not contain all four nucleotides in the ②→3 fragment, and fails the corresponding filter. If M2 extension is penalized less heavily, by setting σ = δ, a longer period of ②→3 alignment is included in the state path during Viterbi decoding, including all four nucleotides and allowing the event to be called as significant. Note that “Anc” refers to the assumed ancestral sequence and “AncC” refers to the complement of this sequence. (TIF) [file pgen.1009221.s009.tif]

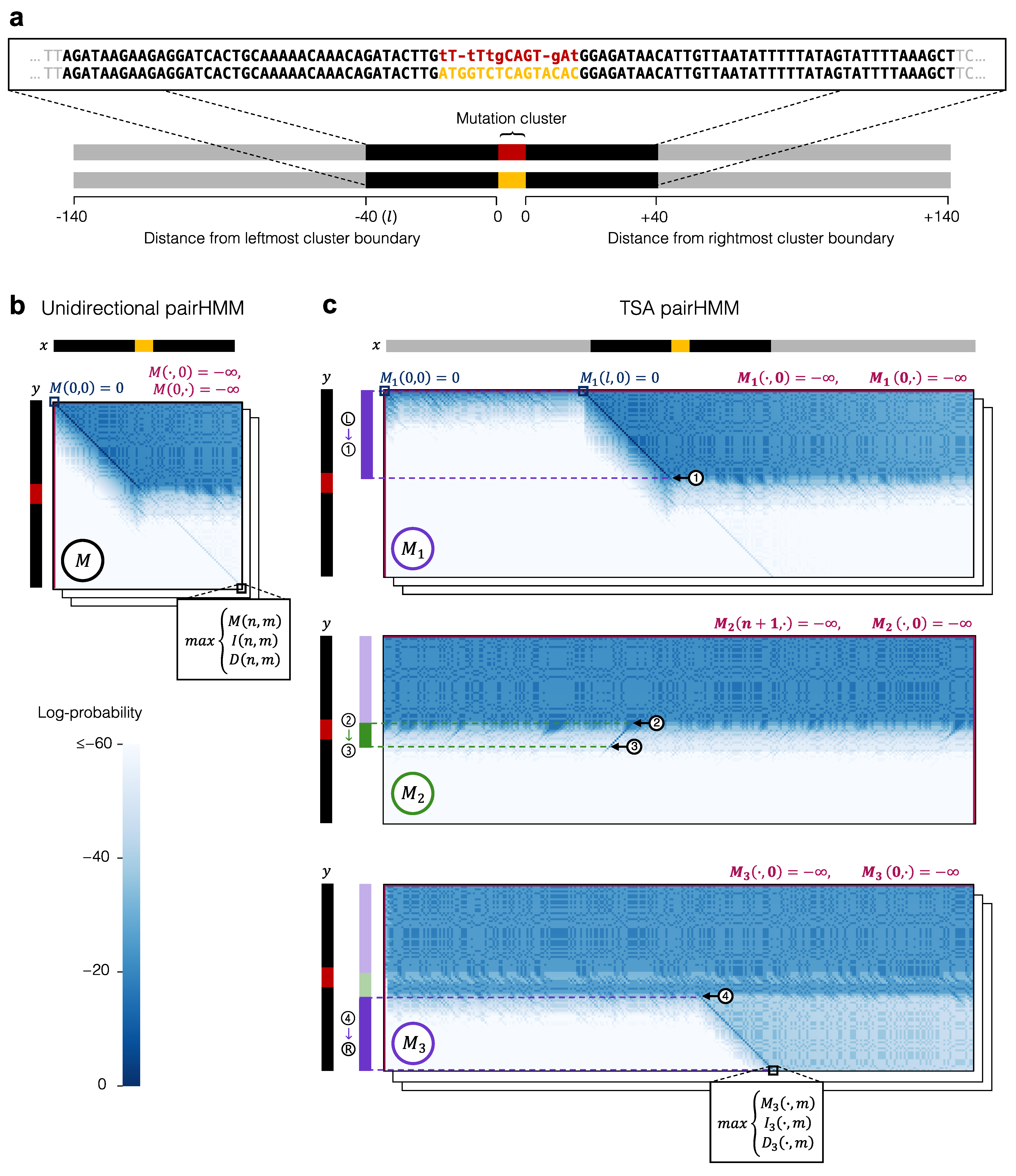

Supplement: S10 Fig — (A) Given an input linear alignment (top), a focal mutation cluster is identified when there are ≥2 substitutions or indel positions within a 10nt window (yellow and red sequence blocks). Mutation clusters vary in their sizes; the 10bp window used for cluster identification is expanded once two differences are found, continuing to expand the rightmost cluster boundary as long as additional differences are found with each iteration of boundary increase. Once a focal mutation cluster is defined (red, yellow), the sequences used for re-alignment are defined separately for each model. (B) For the unidirectional pairHMM, the sequence regions defined by the red/yellow mutation cluster in addition to ±40nt flanking sequence (black, from (A) above) are used for alignment. Unidirectional alignment then follows Algorithm A in S1 Algorithms: the figure illustrates initialisation and subsequent calculation of the M matrix of Algorithm A in S1 Algorithms, omitting the I and D matrices for clarity. (C) For the TSA pairHMM, in addition to the yellow, red and black regions aligned under the unidirectional pairHMM, a further ±100nt region is included for (ancestral) sequence x (grey, from (A) above) to provide additional upstream/downstream search space for the ②→3 fragment. Template switch alignment then follows Algorithm B in S1 Algorithms. For clarity, initialisations and recursive calculations are only illustrated for the match (M) matrices. Note the reverse complement alignment (top right to bottom left) in M2. The unidirectional and TSA pairHMM alignments for this event are given under Event 124 in S1 Data. (TIF) [file pgen.1009221.s010.tif]
