# Supplementary material for: Short-range template switching in great ape genomes explored using pair hidden Markov models
Source: PLoS Genet. 2021 Mar 2;17(3):e1009221. doi: 10.1371/journal.pgen.1009221 (PMC7954356; doi:10.1371/journal.pgen.1009221)
Supplement: S1 Table — Event types are defined by ancestral switch point ordering, and the ensuing rearrangement patterns observed in the descendant sequences. Some pairs of event types are indistinguishable without knowledge of the direction of replication during which an event arose. We indicate these ‘mirror cases’ as pairs in parentheses. Events that can arise through intra-strand switching are indicated by a preceding *. See [15] for further details. (PDF) [file pgen.1009221.s011.pdf]

Proportions of gold standard template switch events corresponding to different event types. Event types are defined by ancestral switch point ordering, and the ensuing rearrangement patterns observed in the descendant sequences. Some pairs of event types are indistinguishable without knowledge of the direction of replication during which an event arose. We indicate these 'mirror cases' as pairs in parentheses. Events that can arise through intra-strand switching are indicated by a preceding *. See (1) for further details.

**Event type**	**Rearrangement pattern**	**Proportion of gold standard events**
(①-④-③-②, *③-②-①-④)	Inverted repeat	0.49
(③-①-②-④, ①-③-④-②)	Inverted repeat with inverted spacer	0.26
(④-①-③-②, *③-②-④-①)	Inverted and direct repeat	0.11
①-③-②-④	Inverted fragment	0.03
③-①-④-②	Two inverted repeats with inverted spacer	0.03
(④-③-①-②, *③-④-②-①),<br>③-④-①-②, *④-③-②-①	Multiple overlapping inverted and direct repeats	0.08

References

1. Löytynoja, A. & Goldman, N. Short template switch events explain mutation clusters in the human genome. *Genome Research* **27**, 1039–1049 (2017).
