# Supplementary material for: Short-range template switching in great ape genomes explored using pair hidden Markov models
Source: PLoS Genet. 2021 Mar 2;17(3):e1009221. doi: 10.1371/journal.pgen.1009221 (PMC7954356; doi:10.1371/journal.pgen.1009221)
Supplement: S2 Table — (PDF) [file pgen.1009221.s012.pdf]

Details of human-specific genomic features used for enrichment analysis.

Genomic feature	Description
Protein coding regions	Regions with a "CDS" feature annotation in GENCODE v33 (1).
Exons	Regions with an "exon" feature annotation within a protein coding region in GENCODE v33.
Untranslated regions	Regions with either a "three_prime_UTR" or "five_prime_UTR" feature annotation, within protein coding regions in GENCODE v33.
Introns	Protein coding transcripts, excluding exons, processed from GENCODE v33.
Intergenic regions	All regions not annotated as being covered by a gene, processed from GENCODE v33.
Pseudogenes	Regions with a "pseudogene" gene type annotation in GENCODE v33.
lncRNA	Regions annotated as long, non-coding RNA, requiring a "transcript" feature annotation in GENCODE v33.
Promoters	-1000nt to -1 nt upstream of the first position of "transcript" feature annotations that are protein coding, processed from GENCODE v33.
Transcription factor binding sites*	The consensus set of clustered transcription factor binding sites for 161 transcription factors across 91 cell types, released by the ENCODE Project v3 (2). Sites were required to have a score >200 and be present in $\geq 5\%$ of cell types ($>4/91$).
EnhancerAtlas enhancers*	Computationally predicted enhancers across 197 tissue/cell types from the EnhancerAtlas 2.0 database (3) in GRCh37 coordinates, converted to GRCh38 coordinates using liftOver. Enhancers were required to be observed in $>20\%$ of tissues (≥ 40), requiring a 50% reciprocal overlap of coordinates.
Super enhancers*	Computationally predicted super enhancer regions across 99 tissues from dbSUPER, requiring each region is observed in $\geq 5\%$ (5/99) of tissues, converted to GRCh38 coordinates using liftOver.
Human accelerated regions	The union of human acceleration region coordinates from the supplementary information sections of (4–7), converted to GRCh38 coordinates using liftOver.
Primate accelerated regions	Primate accelerated regions reported in GRCh37 coordinates from the supplementary information sections of (6, 8). Primate regions corresponding to human, chimpanzee, and gorilla accelerated evolution were kept and converted to GRCh38 coordinates using liftOver.

*These genomic features were processed as in (9).

References

1. Frankish, A. *et al.* GENCODE reference annotation for the human and mouse genomes. *Nucleic Acids Research* **47**, D766–D773 (2019).
2. The ENCODE Project Consortium. An integrated encyclopedia of DNA elements in the human genome. *Nature* **489**, 57–74 (2012).
3. Gao, T. & Qian, J. EnhancerAtlas 2.0: an updated resource with enhancer annotation in 586 tissue/cell types across nine species. *Nucleic Acids Research* **48**, D58–D64 (2020).
4. Prabhakar, S., Noonan, J. P., Pääbo, S. & Rubin, E. M. Accelerated evolution of conserved noncoding sequences in humans. *Science* **314**, 786 (2006).
5. Bird, C. P. *et al.* Fast-evolving noncoding sequences in the human genome. *Genome Biology* **8**, 1–12 (2007).
6. Lindblad-Toh, K. *et al.* A high-resolution map of human evolutionary constraint using 29 mammals. *Nature* **478**, 476–482 (2011).
7. Gittelman, R. M. *et al.* Comprehensive identification and analysis of human accelerated regulatory DNA. *Genome Research* **25**, 1245–1255 (2015).
8. Kostka, D., Holloway, A. K. & Pollard, K. S. Developmental loci harbor clusters of accelerated regions that evolved independently in ape lineages. *Molecular Biology and Evolution* **35**, 2034–2045 (2018).
9. Collins, R. L. *et al.* A structural variation reference for medical and population genetics. *Nature* **581**, 444–451 (2020).
