# Supplementary material for: Short-range template switching in great ape genomes explored using pair hidden Markov models
Source: PLoS Genet. 2021 Mar 2;17(3):e1009221. doi: 10.1371/journal.pgen.1009221 (PMC7954356; doi:10.1371/journal.pgen.1009221)
Supplement: S1 Algorithms — (PDF) [file pgen.1009221.s013.pdf]

Supplementary algorithms

Emission probabilities

As stated in the main text, in the following algorithms match and mismatch emission probabilities are defined under the JC69 substitution model (1), giving for state M:

$$s_M(x_i, y_j) = \begin{cases} \frac{1}{4} + \frac{3}{4}e^{-t} & \text{if } x_i = y_j \\ \frac{1}{4} - \frac{1}{4}e^{-t} & \text{otherwise} \end{cases}$$

where x_i is the ancestral nucleotide, y_j is the descendant nucleotide, and t is the divergence between the two species in the pairwise comparison. The insertion emission probability is $s_I = 0.25$, and the deletion emission probability is $s_D = 1$. In the following, function $comp(y_j)$ simply returns the complement of the input nucleotide y_j, so $comp(A) = T$, $comp(G) = C$, etc.

Identifying mutation clusters and defining sequence regions for re-alignment under each pairHMM

The following procedure is depicted for an example event in S10 Fig. As described in Methods, input to both pairHMMs is determined by scanning a pre-existing pairwise alignment for clusters of mutations, defined as $\geqslant$2 pairwise differences within a 10nt sliding window. Once $\geqslant$2 pairwise differences are identified, an iterative procedure is initiated which extends the rightmost cluster boundary while additional pairwise differences are present. A 10nt region downstream of the current boundary is searched for additional differences; if any are found, the cluster boundary is updated using the position of the rightmost difference. This procedure is repeated until no additional differences are found, defining one focal mutation cluster per candidate template switch event (S10 Fig, a).

With the focal mutation cluster coordinate boundaries established, we define the total input pairwise alignment region to be realigned under our models separately for the unidirectional pairHMM and the TSA pairHMM. The unidirectional pairHMM takes as inputs (x and y) the sequences contained within the pairwise alignment defined by the above cluster boundaries plus a $\pm$40nt flanking region from each sequence (S10 Fig, a & b). (The -40 position, representing the leftmost alignment boundary for the unidirectional pairHMM, is referred to as l below.) This flanking region provides sufficient alignment space to interpret the mutational footprint of a putative template switch event within the context of neutrally evolving sequence that should contain few or no differences. In contrast, the TSA pairHMM realigns this same region but includes an additional $\pm$100nt from the assumed ancestral sequence (x), to provide additional flanking search space for the ②$\rightarrow$③ sequence fragment (S10 Fig, a & c).

To make a fair comparison of the alignments emitted by each pairHMM, despite their using these two different length ancestral sequences, it is necessary to constrain the start and end positions of the TSA pairHMM alignments to match those of the unidirectional pairHMM. This ensures that the flanking region alignments are identical between the two models, and therefore contribute the same scores to each alignment. The score difference between the two models is then derived solely from the contributions of either a linearly aligned mutation cluster, or a region of reverse-orientation template switch alignment. To impose this constraint on the start position of the TSA pairHMM, we initialise matrices M_1 to 0, and I_1 and D_1 to $\log(0.25)$, at positions corresponding to y_0 (i.e. cells indexed $(l,0)$ below). This causes all possible alignments of upstream flanking regions to have low probability, and the Viterbi-like decoding of the optimal state path should always lead back to $(l,0)$ in M_1, I_1 or D_1, facilitating score comparison between the two pairHMMs. To constrain the end TSA position, we require the Viterbi-like decoding of the TSA pairHMM state path to begin at the highest scoring alignment position for y_m (see the Termination condition of Supplementary Algorithm 2).

Algorithm A: Viterbi algorithm for the unidirectional pairHMM

Given two sequences x and y of lengths n and m, respectively, we find their alignment with the highest probability using the following dynamic programming procedure. We represent the i-th entry of sequence x as x_i, and the j-th entry of sequence y as y_j. To facilitate traceback after estimating the highest probability state path, for each cell of M, I, and D, pointer matrices are used to store the moves back to the cell from which each $M(i,j)$, $I(i,j)$, and $D(i,j)$ was derived. After the termination step, the most probable alignment is recovered using the moves stored in these traceback matrices.Note that $\bullet$ indicates index i or j ranging over all possible values from 0 to n or m, as appropriate.

Initialisation:

$M(\bullet,0) = I(\bullet,0) = D(\bullet,0) = M(0,\bullet) = I(0,\bullet) = D(0,\bullet) = -\infty$

$M(0,0) = 0, \quad I(0,0) = D(0,0) = \log(0.25)$

Recursion:

$i = 1,\ldots,n,\ j = 1,\ldots,m:$

$$M(i,j) = \log(s_M(x_i,y_j)) + \max\begin{cases} M(i-1,j-1) + \log(1-2\delta) \\ I(i-1,j-1) + \log((1-\epsilon)(1-2\delta)) \\ D(i-1,j-1) + \log((1-\epsilon)(1-2\delta)) \end{cases}$$

$$I(i,j) = \log(s_I) + \max\begin{cases} M(i-1,j) + \log(\delta) \\ I(i-1,j) + \log(\epsilon + (1-\epsilon)\delta) \\ D(i-1,j) + \log((1-\epsilon)\delta) \end{cases}$$

$$D(i,j) = \log(s_D) + \max\begin{cases} M(i,j-1) + \log(\delta) \\ I(i,j-1) + \log((1-\epsilon)\delta) \\ D(i,j-1) + \log(\epsilon + (1-\epsilon)\delta) \end{cases}$$

Termination:

$E = \max(M(n,m), I(n,m), D(n,m))$

Algorithm B: Viterbi-like algorithm for the TSA pairHMM

As in the unidirectional pairHMM, given two sequences x and y of lengths n and m, respectively, we find their alignment with the highest probability using the following dynamic programming procedure. As described above (and depicted in S10 Fig, a & c) $n > m$ for the TSA pairHMM, and Viterbi-like decoding must include at least one M_2 in the state path. Traceback is facilitated using pointer matrices as above, with moves from $\{M_1,I_1,D_1\}$ to M_2 and from M_2 to $\{M_3,I_3,D_3\}$ also stored as pointers whenever a jump between these matrices produces a more probable move in the state path. Again, $\bullet$ indicates index i or j ranging over all possible values from 0 to n or m, as appropriate.

Initialisation:

$M_1(\bullet,0) = I_1(\bullet,0) = D_1(\bullet,0) = M_1(0,\bullet) = I_1(0,\bullet) = D_1(0,\bullet) = -\infty$

$M_2(n+1,\bullet) = M_2(\bullet,0) = -\infty$

$M_3(\bullet,0) = I_3(\bullet,0) = D_3(\bullet,0) = M_3(0,\bullet) = I_3(0,\bullet) = D_3(0,\bullet) = -\infty$

$M_1(l,0) = 0, \quad I_1(l,0) = D_1(l,0) = \log(0.25)$

Recursion 1:

Find the optimal alignment of fragment Ⓛ → ① by aligning x and y linearly:

$i = 1,\ldots,n,\ j = 1,\ldots,m:$

$$M_1(i,j) = \log(s_M(x_i,y_j)) + \max\begin{cases} M_1(i-1,j-1) + \log(1-2\delta-\theta) \\ I_1(i-1,j-1) + \log((1-\epsilon)(1-2\delta-\theta)) \\ D_1(i-1,j-1) + \log((1-\epsilon)(1-2\delta-\theta)) \end{cases}$$

$$I_1(i,j) = \log(s_I) + \max\begin{cases} M_1(i-1,j) + \log(\delta) \\ I_1(i-1,j) + \log(\epsilon + (1-\epsilon)\delta) \\ D_1(i-1,j) + \log((1-\epsilon)\delta) \end{cases}$$

$$D_1(i,j) = \log(s_D) + \max \begin{cases} M_1(i,j-1) + \log(\delta) \\ I_1(i,j-1) + \log((1-\epsilon)\delta) \\ D_1(i,j-1) + \log(\epsilon + (1-\epsilon)\delta) \end{cases}$$

Recursion 2:
Find the optimal alignment of fragment ② → ③ by emitting y in reverse complement with respect to x, determining the best position to jump from M_1, I_1, or D_1 with c_i:

$i = 1, \ldots, n:$

$$c_i = \max \begin{cases} \max(M_1(i-1, \bullet)) + \log(\theta) \\ \max(I_1(i-1, \bullet)) + \log((1-\epsilon)\theta) \\ \max(D_1(i-1, \bullet)) + \log((1-\epsilon)\theta) \end{cases}$$

$j = m, \ldots, 1:$

$$M_2(i,j) = \log(s_M(x_i, comp(y_j))) + \max \begin{cases} c_i \\ M_2(i-1, j+1) + \log(1-\sigma) \end{cases}$$

Recursion 3:
Find the optimal alignment of fragment ④ → Ⓡ by emitting x and y linearly, determining the best position to jump from M_2 with k_i:

$i = 1, \ldots, n:$

$k_i = \max(M_2(i-1, \bullet))$

$j = 1, \ldots, m:$

$$M_3(i,j) = \log(s_M(x_i, y_j)) + \max \begin{cases} k_i + \log(\sigma(1-2\delta)) \\ M_3(i-1, j-1) + \log(1-2\delta) \\ I_3(i-1, j-1) + \log((1-\epsilon)(1-2\delta)) \\ D_3(i-1, j-1) + \log((1-\epsilon)(1-2\delta)) \end{cases}$$

$$I_3(i,j) = \log(s_I) + \max \begin{cases} k_i + \log(\sigma\delta) \\ M_3(i-1, j) + \log(\delta) \\ I_3(i-1, j) + \log(\epsilon + (1-\epsilon)\delta) \\ D_3(i-1, j) + \log((1-\epsilon)\delta) \end{cases}$$

$$D_3(i,j) = \log(s_D) + \max \begin{cases} k_i + \log(\sigma\delta) \\ M_3(i, j-1) + \log(\delta) \\ I_3(i, j-1) + \log((1-\epsilon)\delta) \\ D_3(i, j-1) + \log(\epsilon + (1-\epsilon)\delta) \end{cases}$$

Termination:

$E = \max(M_3(\bullet, m), I_3(\bullet, m), D_3(\bullet, m))$

References

1. Jukes, T. & Cantor, C. Evolution of protein molecules. In Munro, H. N. & Allison, J. B. (eds.) *Mammalian protein metabolism*, chap. 24, 22–126 (Academic Press, New York, 1969).
